# Association Between Diabetes Mellitus–Tuberculosis and the Generation of Drug Resistance

**DOI:** 10.3390/microorganisms12122649

**Published:** 2024-12-20

**Authors:** Axhell Aleid Cornejo-Báez, Roberto Zenteno-Cuevas, Julieta Luna-Herrera

**Affiliations:** 1 Laboratorio de Inmunoquímica II, Escuela Nacional de Ciencias Biológicas, Instituto Politécnico Nacional, Prolongación de Carpio y Plan de Ayala S/N, Col. Casco de Santo Tomas, Delegación Miguel Hidalgo, Mexico City C.P. 11340, Mexico; axcornejo@uv.mx; 2 Instituto de Salud Pública, Universidad Veracruzana, Av. Luis Castelazo Ayala s/n, A.P. 57, Col. Industrial Animas, Xalapa C.P. 91190, Veracruz, Mexico

**Keywords:** type 2 diabetes mellitus, tuberculosis, *Mycobacterium tuberculosis*, T2DM–TB binomial, innate immune response, drug resistance, efflux pumps, metformin

## Abstract

Tuberculosis (TB), caused by *Mycobacterium tuberculosis* (*Mtb*), remains one of the leading infectious causes of death globally, with drug resistance presenting a significant challenge to control efforts. The interplay between type 2 diabetes mellitus (T2DM) and TB introduces additional complexity, as T2DM triples the risk of active TB and exacerbates drug resistance development. This review explores how T2DM-induced metabolic and immune dysregulation fosters the survival of *Mtb*, promoting persistence and the emergence of multidrug-resistant strains. Mechanisms such as efflux pump activation and the subtherapeutic levels of isoniazid and rifampicin in T2DM patients are highlighted as key contributors to resistance. We discuss the dual syndemics of T2DM–TB, emphasizing the role of glycemic control and innovative therapeutic strategies, including efflux pump inhibitors and host-directed therapies like metformin. This review underscores the need for integrated diagnostic, treatment, and management approaches to address the global impact of T2DM–TB comorbidity and drug resistance.

## 1. Introduction

Diabetes mellitus (DM) is a chronic metabolic disorder characterized by either the insufficient production of insulin by pancreatic beta cells or the ineffective utilization of insulin by the body’s cells, resulting in elevated blood glucose levels and chronic inflammation. Type 2 diabetes mellitus (T2DM) is the most common form of DM, accounting for approximately 90% of cases worldwide [1,2]. Hyperglycemia in T2DM results from the progressive loss of beta cell function [2], increasing the risk of chronic complications such as cardiovascular disease and microvascular damage, including diabetic kidney disease, retinopathy, and neuropathy. These complications can lead to blindness, chronic kidney disease, and a significant reduction in overall quality of life. In 2021, an estimated 537 million adults aged from 20 to 79 were living with diabetes worldwide, equivalent to roughly 1 in 10 individuals. This number is projected to rise to 643 million by 2030 and 783 million by 2045. Furthermore, diabetes was responsible for 6.7 million deaths in 2021. An additional 541 million adults were estimated to have impaired glucose tolerance (IGT), placing them at a high risk of developing T2DM [3].

People with T2DM are more susceptible to infections due to an impaired immune system, which reduces its ability to effectively combat invading pathogens [4,5]. Among the most frequent infections in individuals with T2DM are urinary tract infections [6], skin and soft tissue infections [7,8,9,10] and respiratory infections, including influenza [11,12,13], COVID-19 [14,15], and pneumonia [16,17,18,19,20]. Notably, tuberculosis (TB) is of particular concern in this population, as T2DM significantly increases the risk of developing active TB and exacerbates disease severity [21,22,23].

Declared a global health emergency by the World Health Organization (WHO) in 1993 [24], TB remains a one of the leading infectious causes of death worldwide. This disease is primarily caused by *Mtb*, which typically targets the lungs, but can disseminate to other organs and tissues, such as the lymph nodes, pleura, kidneys, gastrointestinal tract, bones, and central nervous system, causing extrapulmonary TB [25]. Furthermore, TB can persist in a latent form, where *Mtb* remains in a dormant state for extended periods, posing the potential to reactivate and progress to active TB disease. This progression is especially relevant in T2DM patients, whose compromised immune responses further increase their susceptibility to *Mtb* infection and hinder the ability to control bacterial proliferation. Effective host resistance to *Mtb* requires a complex interplay between innate and adaptive immune responses, both of which are impaired in the context of T2DM [26]. In 2022, 7.5 million new TB cases were reported globally—the highest number since the World Health Organization (WHO) initiated global TB surveillance in 1995. That same year, TB caused approximately 1.3 million deaths worldwide, and an estimated 410,000 individuals developed multidrug-resistant TB (MDR-TB) [24]. Several well-established risk factors contribute to the development of TB, including malnutrition, HIV infection, alcohol use disorders, smoking, and T2DM [27]. The increasing prevalence of T2DM in recent years has significantly influenced the epidemiological dynamics of TB, particularly in regions such as Asia and Latin America [24].

The primary aim of this review is to examine the interplay between T2DM and TB, focusing on how T2DM-induced metabolic and immune dysfunction contributes to *Mtb* persistence and drug resistance. It highlights the key mechanisms involved, including the host’s immune response, efflux pump activation, genetic mutations, and altered drug pharmacokinetics, while proposing innovative strategies such as efflux pump inhibitors and host-directed therapies to address the dual burden of T2DM–TB and its role in drug resistance. To form this review, we conducted comprehensive searches in databases such as PubMed, ScienceDirect, and Google Scholar, without imposing any restrictions on language or date of publication. The search included keywords such as “Type 2 Diabetes Mellitus, Tuberculosis, *Mycobacterium tuberculosis*, T2DM–TB binomial, innate immune response, macrophages, drug resistance, efflux pumps, and BCG”, with no restrictions on region or language.

## 2. Epidemiology of the T2DM–TB Binomial

The association between T2DM and TB was a prominent topic in the medical literature during the first half of the 20th century. However, this interest waned with the advent of antibiotics to treat TB and insulin to manage T2DM. The link resurfaced in the late 1990s due to the global rise in T2DM prevalence, particularly in countries where TB is endemic [28]. The WHO recognizes T2DM as a significant risk factor for TB development. However, establishing a clear association between T2DM and TB incidence at the national level remains challenging. This difficulty arises from various confounding factors, including socioeconomic disparities, limited access to healthcare, and the high prevalence of HIV in regions with a substantial TB burden. These factors may either obscure the direct relationship between T2DM and TB or exacerbate their combined impact [24,27]. Six of the ten countries with the highest global DM cases are classified by the WHO as “high burden” TB nations, accounting for approximately 80% of all TB cases worldwide. This overlap underscores the critical intersection of these two major public health challenges [29]. As of 2019, the global prevalence of DM among individuals with TB was estimated to exceed 15%. The presence of T2DM significantly increases the risk of developing TB, compounding the complex interplay between these diseases [30]. The coexistence of TB and T2DM represents a substantial epidemiological challenge, creating a new syndemic that urgently requires coordinated and proactive intervention from healthcare professionals [31].

In South Asia, DM prevalence among TB individuals is notably high, with pooled estimates indicating 21% (95% CI 18.0–23.0). This region accounts for 44% of global TB cases, with India contributing significantly to this burden. Variations in DM prevalence exist across countries, ranging from 11% in Bangladesh to 24% in Sri Lanka. TB patients with DM face nearly double the risk of mortality (OR = 1.74) and treatment failure (OR = 1.65) compared to non-diabetic TB patients. The expected 151% surge in DM prevalence from 2000 to 2025 underscores the urgent need for integrated screening and management strategies to address this dual epidemic [32].

A meta-analysis of 24 observational studies from 15 countries demonstrated a significant association between DM and multidrug-resistant tuberculosis (MDR-TB), with DM nearly doubling the odds of developing MDR-TB (pooled OR = 1.97, 95% CI 1.58–2.45). The association persisted across diverse settings, regardless of country income level, type of DM, diagnostic methods, or study design. These findings highlight the critical need for more robust TB treatment strategies and follow-up care for patients with DM, as well as the importance of DM control in improving TB outcomes and reducing the risk of MDR-TB [33].

Additionally, a systematic review and meta-analysis of 64 studies, involving 299,157 participants, revelated that TB–DM patients had nearly twice the odds of mortality (OR 1.88, 95% CI 1.59–2.21) and relapse (OR 1.64, 95% CI 1.29–2.08) compared to TB patients without DM. Limited evidence also suggested that TB–DM patients are at double the risk of developing MDR-TB (OR = 1.98, 95% CI 1.51–2.60). While substantial heterogeneity was observed among the studies, adjustments for confounders and country income levels explained some of these variations. These findings emphasize the need to evaluate cost-effective interventions to improve TB treatment outcomes in patients with DM, particularly in reducing mortality and MDR-TB risks [34].

As T2DM is being increasingly recognized as a risk factor for TB, its rising global prevalence represents a growing threat to TB control efforts. This highlights the pressing need for integrated strategies to address the dual burden of these diseases [35].

## 3. Peculiarities of *M. tuberculosis*

*Mycobacterium tuberculosis* is an obligated human pathogen; it is a highly specialized pathogen that has co-evolved with humans, making it uniquely adept at establishing long-term infections [36]. Other mycobacterial species are also known to cause TB. These belong to the *Mycobacterium tuberculosis* complex (MTBC) and include species adapted to humans, such as *M. tuberculosis* and *M. africanum*, as well as species adapted to animals, such as *M. bovis*, *M. canettii*, *M. caprae*, *M. microti*, *M. pinnipedii*, *M. orygis*, and *M. mungi*. While these species are genetically related, they differ significantly in host tropism and pathogenicity [37].

The cell wall of *Mtb* is rich in mycolic acids and lipids, which contribute to its impermeability to many antibiotics while also enhancing its resistance to desiccation and oxidative stress. This lipid-rich barrier is crucial for its virulence, as it enables the pathogen to evade host immune responses. [38]. Additionally, during the latency phase of infection, *Mtb* utilizes a variety of effector proteins to evade the host immune system, adapting its lifestyle to persist within granulomas. These are complex, organized structures of immune cells formed by the host as a response to chronic infection [39]. Its slow replication rate, which is unusual for bacterial pathogens, allows it to avoid rapid immune detection and respond dynamically to environmental pressures within the host.

One of the most remarkable features of *Mtb* is its ability to adapt to diverse and hostile conditions within the host. It thrives in the hypoxic environment of granulomas by shifting its metabolism to lipid utilization and entering a non-replicating persistence state [40,41]. This pathogen also employs sophisticated mechanisms to modulate the host immune response, such as inhibiting phagosome–lysosome fusion in macrophages and dampening inflammatory cytokine production [42,43]. Furthermore, *Mtb* has evolved an arsenal of effector proteins that interfere with host cell signaling pathways, promoting survival within macrophages and enabling the pathogen to manipulate host cell death pathways [44,45]. This adaptability not only allows *Mtb* to establish chronic infections, but also contributes to its resilience against therapeutic interventions, posing significant challenges in the treatment of tuberculosis [46].

This topic is vast and multifaceted; however, it will not be addressed in detail within the scope of this review.

## 4. Physiology of the T2DM–TB Binomial

Hyperglycemia promotes the growth and spread of *Mtb* by altering both the host’s metabolism and immune response. From a metabolic perspective, hyperglycemia increases the glucose levels in bodily fluids and tissues, providing a direct energy source for *Mtb* metabolism, which facilitates its replication [47]. Regarding immune function, hyperglycemia significantly impairs key defense mechanisms. It reduces macrophage phagocytic capacity, suppresses oxidative responses, and disrupts granuloma formation, which is critical for containing the infection. Furthermore, hyperglycemia perpetuates a chronic inflammatory state mediated by cytokines such as TNF-α, IL-6, and IL-1β. Rather than enhancing bacterial clearance, this inflammatory state contributes to immune dysfunction [48]. Hyperglycemia also modifies the cellular environment by promoting the formation of advanced glycation end products (AGEs), which interfere with normal cell signaling. Moreover, hyperglycemia disrupts autophagy [49], a fundamental process for eliminating *Mtb* from infected macrophages, allowing the pathogen to persist intracellularly.

One study developed a guinea pig model to examine TB progression in the context of T2DM. Diabetic guinea pigs infected with *Mtb* showed more severe and rapid TB progression, higher bacterial burdens, and heightened proinflammatory responses compared to nondiabetic controls. The study also revealed an exacerbated proinflammatory response in the diabetic guinea pigs, characterized by elevated cytokine and chemokine expressions, including interferon-γ, IL-17A, IL-8, and IL-10 in the lungs and interferon-γ, tumor necrosis factor-α, and monocyte chemoattractant protein-1 in the spleen. While glucose-intolerant guinea pigs showed TB progression like the nondiabetic controls early in infection, disease severity surpassed the controls by day 90. This model closely mimics human TB–T2DM comorbidity, offering insights into disease mechanisms and testing interventions to improve the management of this dual burden [50].

These metabolic and immune impairments explain why individuals with T2DM are at a higher risk of developing pulmonary tuberculosis (P-TB) compared to extrapulmonary TB [51]. The comorbidity of T2DM–TB not only increases susceptibility to infection, but also exacerbates clinical outcomes. T2DM is associated with a 1.69-fold higher risk of anti-TB treatment failure and mortality during treatment and a 3.89-fold greater risk of relapse [52,53]. Furthermore, individuals with T2DM–TB often experience delayed smear conversion during follow-up, prolonging the period of active infection and enhancing TB transmission [31,54].

Notably, recent studies have shown that glycosylated hemoglobin (HbA1c) levels equal to or greater than 7% are a significant risk factor for developing isoniazid resistance (INH-R) and MDR-TB in patients with T2DM–TB [54,55]. This population also frequently presents with more cavitary lesions on chest radiographs, experiences more adverse effects from anti-TB medications, and requires hospitalization more often compared to patients with TB alone [56]. Poor glycemic control in T2DM–TB patients further exacerbates immune dysfunction, increasing their vulnerability to the infectious agent and reducing their response to TB treatment [23,57].

A study conducted in Mashhad, Iran, assessed the prevalence of diabetes among TB patients and investigated the associated factors. A total of 405 individuals diagnosed with TB were screened for diabetes using diagnostic criteria that included HbA1c levels exceeding 6.5%, fasting blood sugar (FBS) levels above 126 mg/dL, or a self-reported diagnosis of T2DM. The study revealed a high prevalence of T2DM among TB patients, with 21.2% of individuals affected—higher than the figures reported in many other countries. Of these cases, 3.5% were newly diagnosed. The study also identified significant associated factors such as age, body mass index (BMI), and TB type [58].

## 5. Innate Immune Response to *M. tuberculosis*

To understand why TB is more readily established and progresses in individuals with T2DM, it is essential to compare the innate immune response between a healthy individual and one with T2DM [59]. When *Mtb* enters the respiratory tract, it encounters the first line of defense in the lungs, composed of airway epithelial cells, macrophages, neutrophils, dendritic cells, natural killer cells, mast cells, and the complement system [60]. This is where *Mtb* initiates a cascade of immune responses by interacting with macrophages.

*Mtb* antigens, both on the cell surface and secreted, manipulate the host’s immune system. Macrophages recognize lipoarabinomannan glycolipids (LAMs) and their lipomannan precursors (LMs) through innate pattern recognition receptors (PRRs) [61], which play a key role in identifying pathogens by detecting pathogen-associated molecular patterns (PAMPs). PRRs activate processes to eliminate infectious agents while promoting anti-tumoral and immunoprotective activities [62,63]. They achieve this by recognizing highly conserved microbial components, including carbohydrates (e.g., lipopolysaccharides, mannose, fructose, and sucrose), nucleic acids (DNA and RNA), and peptides (e.g., flagellin, peptidoglycans, lipoteichoic acids, and muramyl dipeptides) [64]. A crucial subset of PRRs is the toll-like receptors (TLRs), predominantly expressed on antigen-presenting cells (APCs). TLRs on the cell surface recognize bacterial cell wall components, internalize microbes, and trigger an inflammatory response mediated by activated B cells. TLRs located within the endosomal membrane detect microbial nucleic acids and induce type I interferon (IFN) production. Notably, TLR8 expression is upregulated in differentiated macrophages following *Mtb* infection [65,66,67]. Nucleotide-binding oligomerization domain-like receptors (NLRs) are another family of innate immune receptors that detect intracellular pathogens and endogenous by-products of tissue injury. NLRs contribute to various biological processes, including antigen presentation regulation, inflammatory responses, and cell death [64,68,69].

C-type lectin receptors (CLRs) are transmembrane proteins that influence the activation and regulation of phagocytosis in cells such as macrophages, dendritic cells, and neutrophils. CLRs primarily recognize microbial cell wall components and modulate innate immunity by triggering inflammatory and antimicrobial responses. They also detect modified self-antigens, such as damage-associated molecular patterns (DAMPs) released from dead cells [70,71].

The major histocompatibility complex (MHC) is a family of genes that encode specialized receptors essential for antigen presentation between antigen-presenting cells (APCs) and T cells, initiating the adaptive immune response. MHC class I molecules present processed endogenous antigens, while MHC class II molecules present processed exogenous antigens to specialized APCs [72,73]. APCs, such as dendritic cells, macrophages, and B cells, are activated and migrate to the lymph nodes, where they present antigens to T cells. CD4^+^ helper T cells coordinate the immune response, while CD8^+^ T cells destroy infected cells and produce inflammatory cytokines like interferon-gamma (IFN-γ). CD4^+^ T cells play a critical role in macrophage activation through the production of IFN-γ, which directly enhances macrophage functionality to control the infection and facilitates the development of an effective antibody response [73,74]. In individuals with T2DM, CD4^+^ T cells are less effective in producing IFN-γ, compromising the immune system’s ability to control the infection and increasing the risk of chronic or reactivated TB. Another key cytokine in TB control is TNF-α. While macrophages and dendritic cells are the primary producers of TNF-α during infection, CD4^+^ T cells also contribute significantly to its production. TNF-α is essential for maintaining the structure and function of granulomas, which are critical for containing *Mtb* [56]. Additionally, cytokines such as granulocyte-macrophage colony-stimulating factor (GM-CSF), interleukin-1 (IL-1), interleukin-10 (IL-10), and transforming growth factor-beta (TGF-β) play vital roles in modulating the innate immune response during TB infection [75,76]. These cytokines work collectively to regulate inflammation, enhance macrophage activation, and maintain immune homeostasis.

### Phagosome Maturation

In healthy individuals, macrophages play a crucial role in containing *Mtb* by phagocytosing the bacteria and initiating a process known as phagosome maturation. This process, which is essential for the degradation of *Mtb*, involves the acidification of the phagosome and its subsequent fusion with lysosomes.

During phagocytosis, macrophages engulf mycobacteria, forming a phagosome. Once inside the phagosome, the mycobacterium is subjected to a maturation process that enables the fusion of the phagosome with lysosomes. Lysosomes contain a variety of defensins and lytic enzymes, such as lipases, hydrolases, and proteases, which function optimally in an acidic environment with a pH of 4.5 to 5, creating an acidic environment ideal for the degradation of phagocytized particles [42]. The key mechanisms involved in phagosome maturation include phagosome acidification, the generation of reactive oxygen species (ROS) and nitric oxide (NO), and the synthesis of antimicrobial peptides, proteins, and degradative enzymes. Additionally, this maturation process is enhanced by increased chloride ion concentrations and the action of interferon-gamma (IFN-γ) [77].

For most bacteria, internalization and exposure to the acidic and hydrolytically active environment of the phagosome are sufficient to eliminate it, but, *Mtb* has evolved mechanisms to evade this process, allowing it to survive and persist within macrophages. One of the most studied mechanisms is the blocking of normal maturation and the acidification of the phagosome by *Mtb.* The absence of acidification in *Mycobacterium*-containing phagosomes is due to the lack of accumulation of two vacuolar enzymes, ATPase and GTPase. This lack of acidification due to the exclusion of vacuolar ATPase has negative consequences due to the inefficiency of antigen degradation and presentation [42]. Another protein responsible for preventing phagosome–lysosome fusion is coronin 1, which is specific to lymphocytes, macrophages, and neutrophils, the function of which is to keep the plasma membrane bound to the cytoplasm and to integrate signals received from beyond the cell [78]. Coronin 1 is also positively regulated during infection and recruited to phagosomes containing active mycobacteria, where it inhibits lysosome–phagosome fusion by activating a calcium-dependent phosphatase-calcineurin [79,80]. However, when it is retained in the phagosome, there is an absence of coronin 1 in the cytoplasm and, consequently, no activity of calcineurin, which is necessary for phagosome–lysosome fusion [80]. Furthermore, phagocytosis in macrophages activates NDPH oxidase, which catalyzes the production of superoxide, giving rise to reactive oxygen species (ROS) through a series of reactions. Several other ROS are then produced, including hydrogen peroxide, hypochlorous acid, and hydroxyl radicals [43,81]. TNF-α is essential for the microbicidal activity of macrophages; an excess of TNF-α produces susceptibility by increasing mitochondrial ROS (mROS) through reverse electron transport, which initiates a signaling cascade that causes the pathogenic necrosis of *Mtb*-infected macrophages [82,83,84]. On the other hand, IFN-ɣ in macrophages induces the expression of the enzyme iNOS, which catalyzes the production of nitric oxide (NO) radicals, a critical step in the successful control of TB infection [75,84]. In T2DM, chronic oxidative stress alters ROS production, leading to insufficient bactericidal activity. Additionally, interferon-gamma (IFN-γ), which induces the expression of inducible nitric oxide synthase (iNOS) for NO production, is less effective in activating macrophages in T2DM. This impairs the host’s ability to control *Mtb* infection [85].

This inflammatory response persists until the development of an acquired immune response, which is essentially dependent on dendritic cells, since these transport *Mtb* antigens to the lymph nodes, where antigen presentation with T lymphocytes occurs. Once a specific immune response to *Mtb* is acquired, bacterial replication is restricted and the infection enters a state of containment with a relatively static bacterial load [86]. In T2DM, chronic inflammation impairs this process, delaying the development of a robust adaptive immune response and prolonging bacterial replication. The above describes the immune response that occurs in healthy individuals who are exposed to *Mtb*; however, this response depends greatly on the type of mycobacterial lineage, since, as mentioned above, some strains are more pathogenic than others [87,88].

## 6. T2DM Promotes Chronic Inflammation

Individuals with T2DM experience a higher frequency and severity of pulmonary infections, including TB. This increased susceptibility is attributed to immune dysfunction and structural abnormalities caused by T2DM-induced oxidative stress (OS) and chronic inflammation [89]. T2DM is characterized by a state of chronic low-grade inflammation driven by elevated levels of pro-inflammatory cytokines and disrupted signaling pathways, which significantly compromise the immune response against *Mtb.* While inflammation is essential for tissue repair and pathogen elimination, unresolved chronic inflammation becomes detrimental to the host. This condition arises from the persistent production of ROS, proteases, and growth factors by neutrophils and macrophages [90]. Prolonged activation of the innate immune system impairs insulin secretion and action, and this inflammation contributes to macrovascular and microvascular complications of T2DM [91]. When chronic inflammation occurs without microbial involvement, it is referred to as sterile inflammation. This type of inflammation is characterized by the recruitment of neutrophils and macrophages, accompanied by the production of pro-inflammatory cytokines and chemokines, particularly TNF-α and IL-1 β [92]. Some inflammatory markers associated with T2DM, including TNF-α, interleukin-6 (IL-6), IL-1β, and C-reactive protein (CRP), are consistently elevated in T2DM and play critical roles in TB susceptibility and progression [93]. For example, TNF-α, which is essential for granuloma formation and *Mtb* containment, is chronically overexpressed in T2DM. This dysregulation can lead to impaired granuloma maintenance, increased necrosis, and bacterial escape and dissemination. Similarly, IL-6, another cytokine elevated in T2DM, is linked to impaired macrophage function and a reduced production of ROS and reactive nitrogen intermediates (RNIs), both of which are crucial for the intracellular killing of *Mtb* [93,94].

Overweight and obesity, major risk factors for T2DM, further exacerbate this inflammatory state [95]. Obesity is a chronic condition characterized by excessive body fat accumulation, which induces low-grade inflammation that affects multiple organs, activates the innate immune system, disrupts the metabolic balance, and causes tissue damage through increased fibrosis and necrosis [95,96]. Inflammatory processes within pancreatic islets gradually lead to the loss of β-cell mass and dysfunction, culminating in the development of T2DM [97,98,99]. The accumulation of macrophages in adipose tissue, alongside a shift to a pro-inflammatory macrophage phenotype, is closely associated with insulin resistance [100]. Macrophages and adipocytes secrete cytokines such as TNF-α, IL-6, IL-1β, IL-12, IL-18, and IL-23, as well as chemokines like CXC chemokine ligands (CXCL) 1 and 3, which are key drivers of chronic inflammation [101,102]. Among these, IL-6 and TNF-α perpetuate insulin resistance and metabolic dysfunction [100,103]. Elevated CRP levels, another hallmark of chronic inflammation, have been linked to an increased risk of developing T2DM [104]. This interplay between inflammation, immune abnormalities, and metabolic disorders supports the hypothesis that T2DM is a disorder of the innate immune response [105].

Chronic inflammation is directly linked to insulin resistance and the development of T2DM through the activation of the inflammasome [106]. The inflammasome is a multiprotein complex responsible for activating caspases 1, 4, and 5, which process and secrete the proinflammatory cytokines IL-1β and IL-18 [107,108]. Specific inflammasomes, including NLRP1, NLRP3, NLRC4, NLRP6, NLRP7, and NLRP12, belong to the NOD-like receptor (NLR) and AIM2-like receptor (ALR) families [109]. The canonical inflammasome comprises a cytoplasmatic sensor (NLRs and ALRs receptors), the ASC adaptor protein, and an effector caspase (pro-caspase 1). It is activated in response to cellular perturbations caused by pathogen-associated molecular patterns (PAMPs) or damage-associated molecular patterns (DAMPs) and plays crucial roles in pyroptosis (inflammatory programmed cell death), apoptosis, inflammation, and tumor regulation [110,111]. Among these, the NLRP3 inflammasome is particularly critical in regulating immune responses and maintaining homeostasis during pathogen exposure or cellular stress. Found in neutrophils, monocytes, macrophages, dendritic cells, and microglia [112,113], the NLRP3 inflammasome can be activated by pathogens or endogenous danger signals, such as molecules released during tissue injury (e.g., extracellular ATP, hyaluronan, β-amyloid fibrils, lipids, and uric acid crystals) and cellular stress [111]. Upon activation, the NLRP3 receptor protein forms a complex with the ASC protein, which then binds to pro-caspase-1. This complex undergoes self-cleavage that activates caspase-1, leading to the maturation of IL-1β and IL-18 and the activation of the pore-forming protein gasdermin D (GSDMD), resulting in pyroptosis [114,115,116].

The activation of the inflammasome releases of substantial amounts of IL-1β and IL-18, which can damage pancreatic islets, exacerbate insulin resistance, and ultimately contribute to the development of T2DM [117]. IL-1β promotes excessive inflammation and tissue damage, compromising the host’s ability to control *Mtb* infection effectively. Chronic hyperglycemia in T2DM further amplifies these effects by inducing advanced glycation end products (AGEs) and activating their receptor (RAGE), which enhances inflammatory signaling and disrupts macrophage phagosome maturation [102,118].

### Alterations in Innate Immunity Induced by T2DM and Their Role in Susceptibility to M. tuberculosis

T2DM compromises the innate immune response, creating an environment conducive to the establishment and progression of *Mtb* infection. In individuals with T2DM, chronic inflammation, oxidative stress, and structural abnormalities disrupt critical immune mechanisms that would otherwise control *Mtb* [119].

Studies indicate that cell-mediated immunity is compromised in T2DM individuals with TB. Peripheral blood mononuclear cells (PBMCs) stimulated with complex mycobacterial antigens showed a heightened secretion of IFN-γ, TNF-β, and IL-10. In addition, diabetic TB patients exhibited a greater Th2 bias and low Th1:Th2 cytokine ratios, which could explain the more rapid deterioration of the clinical condition [120]. The cytokine expression pattern is classified into the Th1 (IFN, TNF, IL-2, and IL-12), Th2 (IL-4, IL-5, IL-6, IL-9, IL-10, and IL-13), and Th3 (TGF-ß1) cytokines. While Th1 cytokines promote cell-mediated immune responses, Th2 cytokines drive antibody-mediated immunity [105].

Kumar and collaborators investigated the impact of T2DM on CD8 T and NK cell responses in active pulmonary TB. Their study analyzed the basal, antigen-specific, and polyclonal induction of type 1 and type 17 cytokine-producing CD8 T and NK cells, as well as cytotoxic markers, in individuals with active TB and T2DM, in comparison to those without T2DM. The study demonstrated that the individuals with T2DM–TB had relatively higher numbers of CD8 T cells secreting IFN-γ, IL-2, and IL-17F after stimulation with mycobacterial antigens. Moreover, individuals with T2DM–TB had higher numbers of NK cells secreting TNF-α, IL-17A, and IL-17F after stimulation with mycobacterial antigens [121].

Nandy et al. investigated hyperglycemia-induced monocyte signaling using adhesion, migration, and transmigration assays of human monocytes of the THP-1 cell line in the presence of normal (5 mM) and hyperglycemic (10 and 20 mM) blood glucose concentrations without chemokines. The study revelated increased monocyte adhesion to the HUVEC monolayer under hyperglycemic conditions. Moreover, increased monocyte migration, transmigration, and stress fiber responses were observed under hyperglycemic conditions compared to normal glucose concentrations. These results suggest that hyperglycemia is a potent activator of monocyte activity, even in the absence of chemokines [122].

C-type lectins are calcium-dependent carbohydrate-binding proteins capable of interacting with glycoproteins and glycolipids on microbial surfaces [123]. Several C-type lectins recognize mannose- and fucose-rich oligosaccharides, which share structural similarities with glucose. In the immune system, myeloid C-type lectin receptors (CLRs) play a crucial role in modulating or inhibiting phagocytosis, antigenic presentation, and cytokine production [124,125]. Ilyas et al. demonstrated that high glucose levels inhibit the binding of C-type lectin to high-mannose glycoprotein, and that the binding of DC-SIGN (a CLR present on the surfaces of macrophages and dendritic cells) to a fucosylated ligand (blood group B) was canceled when high glucose levels existed. Moreover, complement activation via the lectin pathway was inhibited at high glucose and trehalose concentrations. This raises the possibility that the high glucose conditions typical of DM affect protein–oligosaccharide interactions through competitive inhibition [126].

One of the most important mechanisms for the destruction of mycobacteria is the formation of the phagosome–lysosome complex. Previous studies have shown that individuals with T2DM have a reduced expression of genes encoding vacuolar ATPase (ATP6V1H), which is crucial for acidifying the phagosome–lysosome compartment and facilitating bacterial degradation. Furthermore, a decreased expression of hexokinase 2 (HK2), an enzyme required for aerobic glycolysis in macrophages, and a reduced CD28 expression, necessary for effective T-cell co-stimulation by APCs, have been observed [127]. Disruption of the phagosome also activates the cytosolic NLRP3 inflammasome receptor. The *Mtb* type 1 secretion system (ESX-1) triggers NLRP3 activation, leading to caspase-1 and the activation and secretion of IL-1β and IL-18 [128,129,130]. IL-1β is known to inhibit insulin signaling and glucose uptake by preventing the translocation of type 4 glucose transporters to the cell membrane [131]. The activation of NLRP3 can also induce programmed necrotic death in macrophages infected with *Mtb* [128,130].

Several polymorphisms in genes encoding certain cytokines have been reported to promote the development of TB. The most frequently reported pro-inflammatory cytokines include IL-6 and TNF-α, which play important roles in the early response against *Mtb* and are involved in T2DM [132]. In a study by Lara-Gomez, polymorphisms in IL-6 and TNF-α were analyzed in 30 individuals divided into the following three groups: healthy, TB, and T2DM–TB subjects. The study found that 78% of the individuals had the 174 G/G genotype of IL-6, while 90% and 91% had the -308 G/G and -238 G/G genotypes of TNF-α, respectively. The 174 G/G genotype of IL-6 was observed in individuals with T2DM and increased the risk of developing the T2DM–TB comorbidity five-fold. Analysis of the MDR revealed that the combination of 174 G/G IL-6 and -308 G/G TNF-α in healthy individuals increased the risk of developing this comorbidity up to six-fold, whereas, in individuals with T2DM, this risk increased 14-fold [133].

## 7. Influence of T2DM on the Modification of *M. tuberculosis* Genomes: Drug Resistance Generation

The standard treatment for drug-sensitive TB consists of a six-month regimen with first-line drugs. The intensive phase lasts for two months and includes rifampicin (RIF), isoniazid (INH), pyrazinamide (PZA), and ethambutol (EMB). This is followed by a continuation phase of four months with INH and RIF [134,135]. However, although the treatment of drug-sensitive TB has shown excellent recovery rates, the T2DM condition may aggravate the clinical situation by inducing drug resistance in the patient, particularly to INH and RIF [136]. Drug resistance is strongly associated with glycemic control in T2DM, which may contribute to the development of resistance [137].

Resistance to first-line drugs is very common among patients with T2DM. Drug resistance (DR) is defined as the ability of bacteria to replicate in the presence of a drug. It is specific to a single drug or class of drugs, heritable, and increases the minimum inhibitory concentration (MIC) required to prevent mycobacterial growth and replication. TB-DR is considered when *Mtb* is resistant to at least one first-line drug, such as RIF or INH. Multidrug-resistant TB (MDR-TB), characterized by resistance to both RIF and INH, poses a significant threat to global TB control efforts [138,139].

The development of drug resistance in *Mtb* is primarily due to mutations in the bacterial chromosome, including single-nucleotide polymorphisms (SNPs), insertions, and deletions (InDels). These mutations can alter the pharmacological targets of drugs or produce enzymes that degrade them [135,140]. Additionally, *Mtb* employs “intrinsic resistance” mechanisms to survive antibiotic stress. These mechanisms include cell wall thickening, the activation of efflux pumps, an altered expression of transcriptional regulators, and enhanced DNA repair systems [141].

The serum concentrations of isoniazid (INH) and rifampicin (RIF) are significantly influenced by blood glucose levels, with hyperglycemia causing these concentrations to fall below the therapeutic range. Hyperglycemia alters drug pharmacokinetics by affecting absorption, distribution, metabolism, and excretion processes. Additionally, hyperglycemia-induced vascular changes, such as reduced perfusion to critical organs, may disrupt drug distribution. These effects diminish the bioavailability of anti-TB drugs, reducing their efficacy and potentially leading to subtherapeutic dosing [142].

**Figure 1:**
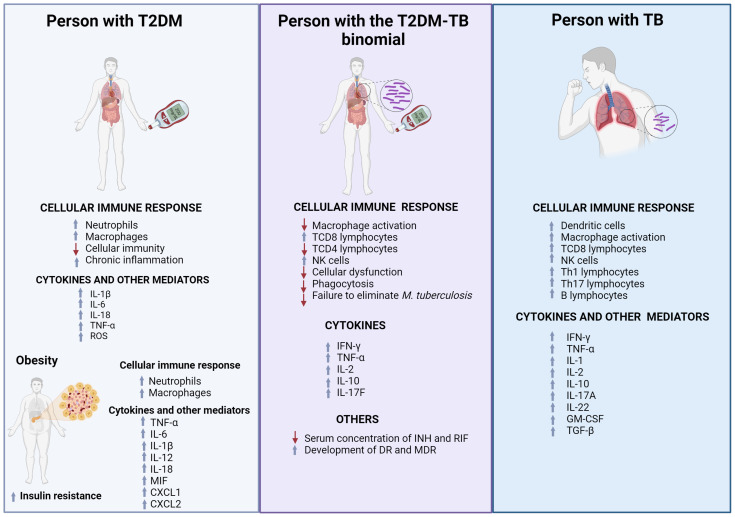
The image presents three cases: one of a person with T2DM, another with TB, and a third with both T2DM–TB simultaneously. This figure illustrates how the immune response is altered in each condition, showing changes in the concentration of immune response cells and cytokines. These changes are represented with blue arrows for increases and red arrows for decreases. Additionally, it highlights that the presence of the T2DM–TB comorbidity promotes the development of DR, which worsens the clinical condition of patients.

This interaction between TB and T2DM creates a vicious cycle, where reduced drug concentrations contribute to treatment failure, an increased bacterial persistence, and the development of drug resistance (DR). The improper administration of anti-TB drugs, exacerbated by glycemic instability in T2DM patients, is one of the most frequent causes of DR [143]. Moreover, T2DM has been implicated in the generation of DR through compensatory mutations in resistance and DNA repair system genes. These mutations are associated with an increased resistance to INH and RIF, further complicating the clinical management of TB in T2DM patients [144,145,146]. Figure 1 summarizes the key points discussed in this review. It presents three scenarios illustrating individuals with distinct medical conditions. The first scenario (on the left) depicts a person with T2DM, the second scenario (on the right) shows a person with TB, and the third scenario (in the center) represents an individual with the T2DM–TB comorbidity. Each sketch highlights the alterations in the immune response and resulting consequences of these conditions, including drug resistance, which is frequently observed in patients with the T2DM–TB comorbidity.

### 7.1. Role of Persistence, Tolerance, and Efflux Pumps Expression in the Development of Drug Resistance

*M. tuberculosis* exhibits phenotypic heterogeneity, characterized by its ability to persist in the presence of antibiotics. This means that, when drug therapy is administered, less than 1% of the population of *Mtb* sensitive to these drugs survives without being genetically resistant. This persistence is clinically significant, because surviving bacteria are more likely to develop resistance when exposed to prolonged drug pressure [147]. Persistent bacteria often enter a state of growth arrest to endure stressful environmental conditions, including those induced by bactericidal drugs. These bacteria exhibit slower death kinetics in response to drug exposure, producing a bimodal death curve [148,149,150].

A bacterium is considered to be persistent or tolerant depending on its population context. A single bacterium with survival advantages is deemed as persistent, while a subpopulation of bacteria exhibiting collective survival advantages is considered as tolerant [150]. Tolerance refers to the ability of bacterial cells to withstand exposure to bactericidal drugs without altering their minimum inhibitory concentration (MIC) [149]. This phenomenon, often linked to treatment failure and relapses, is critical in many bacterial infections, including TB [151].

*Mycobacteria* can develop different mechanisms to tolerate stress conditions, and one of these mechanisms is the expression of efflux pumps. Efflux pumps are membrane-bound transport proteins that expel antibiotics and other toxic compounds from cells, reducing intracellular drug concentrations and enhancing bacterial survival. These pumps are particularly active during environmental stress, such as the antibiotic exposure or nutrient deprivation conditions commonly encountered in the host environment during TB infection [152,153]. 

In *Mtb*, efflux pumps decrease the intracellular concentrations of first-line drugs such as INH and rifampicin RIF, impairing their bactericidal efficacy. This subtherapeutic drug exposure allows *Mtb* to persist during treatment, increasing the likelihood of mutations that confer genetic resistance. As such, efflux pump activation is a critical factor in the emergence of MDR-TB, defined by resistance to both INH and RIF [154,155].

Building on this understanding, this study highlights the combined contribution of drug efflux pumps and mutations in target genes to the drug resistance observed in MDR-TB clinical isolates. Researchers analyzed the expression of key resistance-associated genes (*rpoB*, *katG*, *inhA*, and *oxyR-ahpC*) and six efflux pump genes (*efpA*, *Rv1250*, *Rv1634*, *Rv2459*, *drrA*, and *drrB*) in 27 MDR-TB isolates. Notably, the overexpression of efflux pump genes was observed under isoniazid (INH) and rifampin (RIF) stress, especially when combined with fresh pomegranate juice, signifying their role in resistance. Additionally, a significant downregulation of *katG* and upregulation of *rpoB* were found in most isolates, reinforcing the interplay between genetic mutations and efflux mechanisms in resistance development. These findings challenge the prevailing focus on gene mutations alone and underscore the need to account for efflux pump activity in understanding and managing MDR-TB [156].

### 7.2. Isoniazid Resistance (INH-R)

Isoniazid, known chemically as isonicotinic acid hydrazide, is a synthetic pro-drug used as an anti-tuberculosis drug since 1952. The bactericidal activity of INH is selective and specific for mycobacterial species and the *Mtb* complex [157]. The bactericidal effect of INH depends on the enzyme catalase-peroxidase of *Mtb*, which is encoded by the *katG* gene. Catalase-peroxidase converts INH into the isonicotinyl acyl radical (active form of the drug), which is a potential inhibitor of the ennoyl-acyl reductase transporter protein (InhA) and β-ketoacyl-acyl transporter synthase protein (KasA), two key enzymes for the biosynthesis of mycolic acids, an essential component of the cell wall of mycobacteria, triggering their death [158,159]. Research has shown that *Mtb* resistance against INH is mediated primarily by mutations in several genes, including *katG*, *inhA*, and *fabG1*, as well as the upregulation of INH inactivators or efflux pumps [160,161]. INH-R typically emerges early in new patients during first-line treatment. Furthermore, INH-R is regarded as the initial stage in the development of MDR-TB and XDR-TB [143].

*Mtb* strains with a *katG* genetic deletion or mutation acquire resistance to INH [162]. Two of the most common mutations that lead to resistance to INH are mutations of the *katG* gene, Ser315Thr, which results in a high level of resistance, and the *inhA*-15 mutation, which results in a low level of resistance to INH [141,163]. Mutations in the *Rv1908c* gene, which encodes the mycobacterial enzyme KatG, can also confer resistance to INH. However, these mutations may also be the result of variations in the *fabG1-InhA* genes, which improve the expression of the *inhA* gene. Mutations in this gene cause a decrease in the affinity for NADH [163]. Other resistance mechanisms have been discovered in the *W341R KatG* and *L398P KatG*, genes that confer resistance to INH in *Mtb*, while *R146P KatG* was not found to affect the susceptibility to INH of *Mtb* [164].

Lyu conducted a meta-analysis to assess the association between HbA1c and DR with first-line drugs in patients with T2DM and TB. The study included 390 patients divided into two groups, the first of which included 123 individuals with HbA1c < 7%, while the second comprised 267 patients with HbA1c ≥ 7%. The study found that T2DM–TB patients with HbA1c levels < 7% had a lower risk of developing isoniazid resistance, rifampicin resistance, and MDR. The classification of HbA1c was, thus, identified as a risk factor for the development of isoniazid resistance and MDR [57]. Mutations at the *KatG315N* site are more common in MDR-TB patients alone, while T2DM–TB-MDR patients are more likely to have mutations at the *inhA-15* site [165]. 

Another factor that merits consideration in terms of the generation of DR is the rate of the acetylation of INH. Clinical evidence indicates that individuals with TB who are rapid acetylators are more susceptible to microbiological failures and relapses compared to slow acetylators [166]. The pharmacokinetic variability of INH alone leads to greater microbiological failure, relapses, and acquired DR. Individualized dosing for TB may, therefore, be more effective than the standardized dosing prescribed in directly observed therapy programs [167,168]. The initial stage of INH metabolism involves hepatic acetylation; however, clinical evidence indicates that individuals with T2DM may develop liver failure due to a process involving fat accumulation, chronic inflammation, and liver fibrosis, which leads to liver damage [169] This may, in turn, significantly affect the INH acetylation process and lead to further liver damage [170], and acts to generate a low proportion of the isonicotinyl acyl radical, which is the active form against *Mtb*, promoting resistance to INH in *Mtb* [171]. A study by Pérez-Martínez et al. compared the presence of SNPs in genes related to DNA damage repair (GRDDR) in drug-sensitive and drug-resistant *Mtb* genomes isolated from patients with and without T2DM. The results showed that, from a selection of 63 *Mtb* GRDDR, SNPs were found to exist in *RecR*, *LigB*, and *DnaE1* as the genes associated with the presence of T2DM in the host. Regarding drug-resistant and drug-sensitive isolates, the presence of non-synonymous SNPs was demonstrated in the *RecGwed* gene, which had the highest discriminant value, followed by *MutY*, *DnaE2*, and *Mfd*, demonstrating their high frequency in genomes with a certain level of DR and confirming their utility as discriminants. It is, therefore, possible to determine the presence of DR and T2DM in the host through analysis of these genes [146].

### 7.3. Rifampicin Resistance (RIF-R)

RIF acts on the *rpoB* gene, which codes for the β subunit of RNA polymerase, and most of these mutations are found within the rifampicin resistance determinant region (RRDR) of 81 bp, which encompasses codons 507 to 533. This produces conformational changes that determine a low affinity for the drug and the consequent development of resistance [172,173]. Most mutations to rifampicin occur in three groups in the *rpoB* gene, which encode different amino acids. In *M. tuberculosis*, these mutations are found in group I in the central region (RRDR). The most common mutations occur in codons 531, 526, and 516. In codon 531, Serine is replaced by leucine; in codon 526, histidine is replaced by tyrosine; and in codon 516, aspartic acid is replaced by the amino acid valine [173,174]. In a few cases, mutations have also been detected in the N-terminal region of the rpoB gene, as well as in the *rpoA* and *rpoC* genes encoding the α and β subunits of RNA polymerase [175]. 

Several studies have shown that people with T2DM and TB present a higher risk of MDR-TB, as well as a longer time to sputum conversion [176]. A pharmacokinetic study found that the plasma levels of rifampicin were 53% lower in people with T2DM. This could impact treatment outcomes and generate the emergence of RIF-R strains [136,167]. 

Metwally et al. conducted a meta-analysis to evaluate the impact of T2DM on the pharmacokinetics of RIF. That study revealed that the time to reach the maximum concentration (Tmax) of RIF was increased in T2DM patients compared to non-diabetic patients. The negative impact of T2DM on intestinal motility may be responsible for this result, and hyperglycemia also affects the duodenal P-glycoprotein efflux pump and delays gastric emptying time, which decreases the absorption rate and delays the absorption of RIF in T2DM–TB subjects [177]. Metha et al. conducted a cross-sectional study with 315 adult TB patients with TB and T2DM–TB in south India, which evaluated the association between T2DM and RIF-R among patients with active TB. The results showed that 27.3% of patients with T2DM had RIF-R, compared to 8.8% of patients without T2DM [178]. 

In contrast, Perea-Jacobo conducted a study comparing the serum RIF concentrations in patients with TB and those with T2DM–TB using high-performance liquid chromatography (HPLC). The sample included 30 patients, 14 with TB and 16 with T2DM–TB. The results showed that the highest concentrations of RIF (3.5 and 2.64 µg/mL, respectively) were recorded 2.5 h after ingestion in both groups. The maximum difference in RIF concentration (Cmax) between the TB and T2DM–TB groups was not significant. However, the analysis revealed suboptimal Cmax levels in both groups. This working group, therefore, suggested considering an increase in the current dose of RIF from 10 mg/kg to 15 or 20 mg/kg to enhance its Cmax and, thus, its bactericidal activity [179].

Bermudez-Hernández et al. evaluated DR in diabetic patients, finding mutations in the *rpoB* gene characteristic of RIF-R, in which they detected the four following specific variants of the *rpoB* gene: *H445L*, *S441L*, *Q432P* and *Q432L.* These mutations have a low prevalence and have been classified as SNPs associated with phenotypic resistance against RIF [145].

Lin et al. investigated the relationship between TB and T2DM in the development of genetic mutations associated with MDR. This study analyzed a total of 763 patients, divided into the following two groups: TB and T2DM–TB patients. The results showed that MDR occurred in 32 out of 525 patients in the TB group, and extreme MDR (XMDR) occurred in 15 out of 207 patients in the T2DM–TB group. In the T2DM–TB-MDR group, mutations were mainly observed at the *ropB531* and *ropBS531L* sites, which confer RIF resistance. The most frequent mutations conferring INH resistance in MDR-TB patients were found at the *KatG315N* site, while T2DM–TB-MDR patients were more prone to mutations at the *inhA-15M* site [165].

A recent study developed by Bermudez-Hernández evaluated the influence of T2DM on the dynamics of polymorphisms related to antibiotic resistance in TB. This involved sequencing the *Mtb* genomes of 50 individuals newly diagnosed with TB and with T2DM–TB at days 0 (TB diagnosis), 30, and 60. After gene analysis, the most frequent mutations identified were *katG* S315T, which confers resistance to INH, and *rpoB* S450L, which confers resistance to RIF. In addition, an INH-R isolate was observed to develop a set of pre-XMDR-related mutations in as little as 30 days. Although preliminary, this study shows the accelerated selection process of mutations in resistance genes in individuals with TB–T2DM [180]. 

## 8. Potential Adjunctive Therapies for the Management of the T2DM–TB Comorbidity

### 8.1. Efflux Pumps Inhibitors

From a clinical perspective, understanding and targeting efflux pumps is essential in improving treatment outcomes in TB-DR. Efflux pump inhibitors (EPIs) offer a promising adjunctive strategy to enhance the efficacy of existing anti-TB drugs. By blocking efflux pump activity, EPIs increase intracellular drug concentrations, restoring drug effectiveness and reducing the survival of persistent and tolerant bacterial populations [181,182]. This approach could be especially beneficial for patients with T2DM, who frequently exhibit subtherapeutic drug levels due to hyperglycemia-induced pharmacokinetic alterations [177].

The interplay between T2DM and TB-DR further complicates treatment, as the subtherapeutic concentrations of INH and RIF in T2DM–TB patients create an optimal environment for efflux pump activation and the evolution of drug resistance. Addressing this issue requires a comprehensive treatment strategy that accounts for the metabolic and immune dysregulation inherent in T2DM. This may involve optimizing glycemic control to improve drug bioavailability, incorporating EPIs, and closely monitoring resistance development [183,184]. Efflux pump activity also underscores the need for novel diagnostic tools to detect tolerance mechanisms in *Mtb* populations before the onset of full genetic resistance. The early identification of efflux pump activation could enable personalized treatment regimens, mitigating the risk of TB-DR and improving clinical outcomes [181,185]. 

The relevance of efflux pump activation is particularly pronounced in individuals with T2DM. As previously noted, the serum concentrations of INH and RIF often fall below the MIC in T2DM patients, fostering bacterial survival and promoting tolerance mechanisms like efflux pump activation. These subtherapeutic drug levels, coupled with the metabolic and immune dysregulation associated with T2DM, may accelerate the development of drug-resistant strains [186].

In a study by Gupta et al., they showed that the efflux pump inhibitor verapamil significantly enhanced the bactericidal activity of bedaquiline against *Mtb* in a mouse model. By coadministering verapamil with suboptimal doses of bedaquiline, the researchers achieved the same antimicrobial efficacy as full human-equivalent doses, potentially reducing the drug’s dose-dependent toxicities. Verapamil also inhibited the development of bedaquiline-resistant mutants, indicating its protective role during monotherapy. These findings suggest that verapamil can potentiate bedaquiline’s activity by improving drug retention through efflux pump inhibition, offering a promising strategy to enhance multidrug-resistant tuberculosis treatment while minimizing adverse effects [187].

Despite the clinical importance of this interaction, few studies have explored the role of efflux pump activation in the T2DM–TB binomial. Closing this knowledge gap is crucial for improving the clinical management of these patients and designing therapies that effectively target both persistence and efflux pump activity.

### 8.2. Metformin

There is growing evidence supporting the role of metformin in the prevention and treatment of TB [188]. Metformin is a medication used to treat diabetes that has several host-directed therapeutic effects, including increased autophagy, reduced inflammation, and improved immune cell function [189,190,191,192]. A retrospective cohort study conducted by Lee et al. included 499 patients diagnosed with culture-positive P-TB, with 105 of these patients diagnosed with T2DM. Of these patients, 62 were treated with metformin. The treatment had no significant effect on sputum culture conversion; however, it did improve the sputum culture conversion rate in patients with cavitary P-TB and T2DM, who typically present higher bacterial loads. Metformin may, therefore, be an effective adjunctive anti-tuberculous agent to improve sputum culture conversion after 2 months of treatment [193]. In another study conducted in Taiwan, 5,846 close contacts of TB patients were analyzed. These included metformin users, non-metformin users, and healthy contacts. The incidence of active TB was 755, 1117, and 526 cases per 100,000 people in each group, respectively. The healthy contacts exhibited the lowest risk of developing active TB and metformin use partially reversed the risk associated with T2DM [194].

Furthermore, a phase IIB clinical trial by Kumar et al. conducted a thorough pharmacokinetic study to assess the impact of metformin on the plasma levels of INH, RIF, and PZA in non-diabetic patients with first-line drug-sensitive TB-P. The patients were divided into the following two groups: Group 1 received anti-TB treatment, while Group 2 received anti-TB treatment and metformin. The study found that the patients of Group 2 experienced significant increases in the clearance of rifampicin, isoniazid, and pyrazinamide, relative to Group 1. However, there was little effect on sputum conversion at the conclusion of treatment [195]. Due to the similarities between the metabolic alterations of T2DM and TB, metformin is now under consideration as a possible adjuvant in the pathology of TB. This treatment has the potential to reduce the severity of TB infection in patients with and without T2DM.

## 9. Advances in Diagnostics and Immunization Strategies for Binomial T2DM–TB Control

Traditional phenotypic drug susceptibility tests, such as the BACTEC MGIT 960 system and the Löwenstein–Jensen proportion method, face significant limitations due to the slow growth rate of *Mtb* [196,197]. These delays in resistance detection can compromise timely therapeutic decisions and increase the risk of treatment failure. In contrast, molecular tools like MUBII-TB-DB provide a faster and more efficient alternative by directly identifying the key mutations associated with drug resistance. The MUBII-TB-DB database is a simple, highly structured text-based system that compiles *Mtb* mutations (DNA and protein) occurring at the following seven loci: *rpoB*, *pncA*, *katG*, *mabA(fabG1)-inhA*, *gyrA*, *gyrB*, and *rrs.* This method enables the precise characterization of resistant strains by targeting critical genes, facilitating the management of multidrug-resistant TB [198]. By integrating molecular diagnostics, this approach supports the development of personalized treatment strategies, improving patient outcomes and advancing global TB control efforts.

Regarding prevention, vaccination against TB with the Bacillus Calmette–Guérin (BCG) vaccine is primarily aimed at preventing infection with *Mtb* or the progression to active TB. A study by Radhakrishnan et al. demonstrated significant benefits of BCG vaccination in a mouse model of T2DM infected with *Mtb.* While BCG vaccination did not significantly reduce the bacterial load in the lungs of diabetic mice, it markedly decreased lung inflammation and mortality compared to unvaccinated mice. Notably, the BCG-vaccinated mice exhibited an expansion of IL-13-producing regulatory T cells (Tregs) expressing CXCR3, which facilitated the conversion of proinflammatory M1 macrophages to anti-inflammatory M2 macrophages. This immunological shift reduced excessive inflammation, highlighting a critical mechanism by which BCG mitigates the heightened inflammatory response seen in TB-infected diabetic mice [199]. BCG immunization also influences lipid metabolism and may contribute to the protection afforded against TB by pathways other than those directly involved in immune responses [35]. These findings underscore the vaccine’s potential to modulate immune responses in comorbid conditions, offering therapeutic insights for TB control in vulnerable populations [199]. On the other hand, an emerging modality for the treatment of TB is therapeutic vaccines, which are primarily intended to act as adjunctive treatments to standard therapy, reduce disease recurrence, prevent the reactivation of latent infections, and shorten the duration of therapy to improve treatment adherence. These vaccines, including candidates such as *M. vaccae* (MV), RUTI, and recombinant vaccines such as VPM1002, are designed to modulate host immunity against *Mtb*. For example, MV and RUTI have demonstrated in preclinical studies and small clinical trials the ability to reduce bacterial load, improve sputum culture conversion, and decrease pulmonary pathology. These vaccines act by stimulating specific immune responses, such as CD4^+^ and CD8^+^ T-cell activation, in addition to promoting a more favorable immune environment to combat both replicating and latent bacteria [200]. Therapeutic vaccines have a potentially transformative role in the fight against MDR-TB by improving the efficacy of antimicrobial regimens and providing an additional tool for the overall control of the disease.

## 10. Conclusions

Type 2 diabetes mellitus (T2DM) significantly increases susceptibility to tuberculosis (TB) by weakening the innate immune response, making individuals three times more likely to develop *Mtb* infection. Additionally, T2DM exacerbates the emergence of MDR strains, further complicating treatment and control efforts. Addressing the T2DM–TB syndemic requires a comprehensive and multidisciplinary approach. Healthcare systems should prioritize the early detection of T2DM in TB patients, particularly in those with known risk factors, to ensure timely intervention. Genomic surveillance systems must also be implemented to identify drug-resistance-related mutations, enabling personalized treatment regimens and reducing the transmission of MDR strains.

Adjunctive therapies should be incorporated into treatment strategies to address the complex interplay between T2DM and TB. Therapeutic vaccines have shown potential in modulating immune responses and improving treatment outcomes, while efflux pump inhibitors can restore the efficacy of first-line anti-TB drugs by blocking drug expulsion mechanisms. Metformin, a widely used antidiabetic drug, offers additional promise as a host-directed therapy by alleviating metabolic stress and enhancing immune function. These interventions could significantly improve treatment success and reduce the risk of drug resistance.

Furthermore, public health initiatives must emphasize the importance of maintaining a healthy lifestyle to prevent the onset of T2DM and its associated impact on TB incidence. By integrating metabolic health management, innovative diagnostic tools, tailored therapeutic regimens, and adjunctive therapies, this comprehensive strategy provides a pathway to improving outcomes for T2DM–TB patients, combating drug resistance, and reducing the global burden of these intertwined diseases.

## Data Availability

Not applicable.
